# Postdiagnosis Statin Use and Breast Cancer Mortality

**DOI:** 10.1001/jamanetworkopen.2025.38737

**Published:** 2025-10-30

**Authors:** Sixten Harborg, Lars Pedersen, Henrik T. Sørensen, Thomas P. Ahern, Deirdre Cronin-Fenton, Signe Borgquist

**Affiliations:** 1 Department of Oncology, Aarhus University Hospital/Aarhus University, Aarhus, Denmark; 2 Department of Clinical Epidemiology, Aarhus University/Aarhus University Hospital, Aarhus N, Denmark; 3 Department of Nutrition, Harvard T.H. Chan School of Public Health, Boston, Massachusetts; 4 Center for Population Medicine, Aarhus University, Aarhus C, Denmark; 5 Department of Surgery, Larner College of Medicine, University of Vermont, Burlington; 6 Department of Oncology, Clinical Sciences, Lund University, Lund, Sweden

## Abstract

**Importance:**

Emerging evidence suggests that cholesterol plays a role in breast cancer (BC) metabolism, raising the possibility that cholesterol-lowering medications, such as statins, may improve BC prognosis.

**Objective:**

To evaluate the association between postdiagnosis statin initiation and BC mortality using an emulated target trial approach.

**Design, Setting, and Participants:**

This observational cohort study using a target trial framework included 96 924 women diagnosed with stage I to III BC from 2000 to 2021 from Nationwide Danish registries, including the Danish Breast Cancer Group’s clinical database and the Danish National Prescription Registry. Women with prior invasive BC or prediagnosis use of cholesterol-lowering medication were excluded. Eligible patients were duplicated into a cloned cohort and assigned to 1 of 2 treatment strategies: statin initiation within 36 months postdiagnosis or no statin initiation. Patients were followed up until deviation from the assigned strategy, emigration, death, 10 years of follow-up, or October 5, 2022.

**Exposures:**

Initiation of any statin within 36 months after BC diagnosis.

**Main Outcomes and Measures:**

The primary outcome was BC mortality. Secondary outcomes included all-cause mortality. Hazard ratios (HRs) and 95% CIs were estimated using inverse probability of censoring weighted (IPCW) Cox regression models with a robust variance estimator.

**Results:**

A total of 66 952 patients with BC were enrolled in the emulated target trial (17 152 [27.6%] were aged 50-59 years); 4851 (7.2%) initiated statins within 36 months after diagnosis. Over 606 266 person-years of follow-up, 9130 patients died from BC and 19 679 from any cause. The 10-year risk of breast cancer mortality was 11.8% among statin initiators and 13.5% among noninitiators, corresponding to a risk difference of 1.7% (95% CI, 0.5% to 3.0%). A similar difference was observed for all-cause mortality (23.3% vs 24.5%; risk difference, 1.2%; 95% CI, –0.1% to 2.5%). IPCW analysis yielded an HR of 0.90 (95% CI, 0.85 to 0.95) for BC mortality and 0.92 (95% CI, 0.85 to 1.00) for all-cause mortality among statin initiators vs noninitiators.

**Conclusions and Relevance:**

In this cohort study, among 66 952 women with early BC, postdiagnosis statin initiation was associated with a modest reduction in BC and all-cause mortality. These findings support further investigation into the potential role of statins as an adjunct to standard adjuvant BC treatment.

## Key Points

**Question:**

Is postdiagnosis statin initiation associated with breast cancer mortality?

**Findings:**

In this cohort study emulating a target trial using Danish registry data from 66 952 patients with breast cancer, statin initiation within 36 months postdiagnosis was associated with lower 10-year breast cancer mortality (11.8% among those who received statins vs 13.5% among those who did not; absolute reduction, 1.7 percentage points), and all-cause mortality was 23.3% among those who received statins vs 24.5% among those who did not.

**Meaning:**

These findings suggest that postdiagnosis statin use may provide a clinically meaningful survival benefit in breast cancer patients, with 59 patients needing treatment to prevent 1 additional breast cancer death at 10 years, supporting further randomized trials.

## Teaser

This cohort study evaluates the association between postdiagnosis statin initiation and breast cancer mortality using an emulated target trial approach.

## Introduction

Statins are cholesterol-lowering medications that function by blocking the mevalonate pathway through inhibition of 3-hydroxy-3-methylglutaryl-coenzyme A reductase.^1^ The mevalonate pathway is crucial for the biosynthesis of cholesterol and other isoprenoids, which are essential for various cellular processes, including cell membrane integrity and protein prenylation.^1^ Statins are widely prescribed to prevent cardiovascular events, such as myocardial infarction and stroke, and are known for their efficacy, tolerability, and affordability.^2,3^ Beyond their cholesterol-lowering properties, statins have anti-inflammatory, immunomodulatory, and antiproliferative effects, and therefore are candidates for anticancer therapy.^4^ Preclinical studies have highlighted the major role of cholesterol in breast cancer (BC) metabolism and suggested that elevated cholesterol might contribute to tumor progression.^5,6,7,8,9^ Furthermore, evidence from preclinical studies suggests that statins inhibit cancer cell proliferation, induce apoptosis, and mitigate metastasis in BC models.^10,11^ These preclinical findings spurred numerous epidemiological studies that explored the potential protective effects of statins on BC outcomes and demonstrated a positive association between statin use and improved cancer survival.^12,13,14,15,16,17,18,19,20,21^

The potential protective role of statins in BC gained prominence after a Danish nationwide prospective cohort study indicated that patients with early-stage BC who are prescribed simvastatin have diminished recurrence risk.^13^ A cohort study, including a more recent cohort of BC patients treated with aromatase inhibitors, revealed similar associations.^14^ In a smaller Swedish study, statins were associated with lower risk of distant recurrences.^15^ The association of statins with BC survival has been studied further in the large randomized, phase 3, double-blind Breast International Group 1–98 trial,^18^ including 8010 patients with BC. This trial associated incident statin use during endocrine therapy with 21% improvement in disease-free-survival.^18^ Additionally, a large nationwide Swedish study, including 20 599 Swedish women with BC, associated use of statins postdiagnosis with 17% lower risk of BC-related mortality and 11% lower risk of all-cause mortality.^16^ A similar reduction was observed in a recent meta-analysis of 34 observational studies, including nearly 690 000 women with BC, which found statin use to be associated with lower risks of BC mortality and recurrence, especially among users of lipophilic statins and patients with estrogen receptor-positive disease.^22^ Finally, Emilsson et al^23^ demonstrated that many studies examining the relation between statins and survival have been affected by immortal time bias and reported a null effect of statins on survival in patients with BC. However, the study by Emilsson, et al was limited by a relatively small sample size of 6053 patients with BC and a short follow-up period of a maximum 39 months for cancer-specific mortality.

Physicians often discontinue statins after BC diagnosis because of a misconception that they are irrelevant in the context of malignant disease.^24^ Notably, the second most common cause of death among BC survivors is cardiovascular-related death.^25^ Given the substantial prolongation of survival in patients with early BC and the 32% increased risk of cardiovascular disease in this population,^26^ many patients will face cardiovascular disease, thus increasing the demand for cardioprotective initiatives.^27^

In the current study, we used data from electronic health care records to emulate the ongoing Mammary Cancer Statin ER Positive (MASTER) study,^28^ a randomized, multicenter, double-blind, placebo-controlled phase 3 trial assessing the addition of atorvastatin to standard adjuvant therapy in patients with early BC. We hypothesized that the addition of statins to standard adjuvant therapy in BC would decrease the risk of BC mortality to a greater extent than standard adjuvant therapy alone.

## Methods

For this cohort study, we conducted a nationwide population-based emulation of a target trial using Danish clinical and administrative registries. This study was approved by the Danish Breast Cancer Group and the Danish Data Protection Agency and adheres to the Agency’s General Data Protection Regulations.^29^ The study is based on routinely collected registry data and therefore, according to Danish regulations, does not require separate ethical approval or informed consent. The study is reported in accordance with Strengthening the Reporting of Observational Studies in Epidemiology (STROBE) reporting guidelines for cohort studies.^30^

### Study Setting

We conducted this study in Denmark, where residents have unlimited access to tax-funded health care through the Danish National Health Services.^31^ At birth or during immigration, a unique personal identification number is assigned to all Danish residents. The unique personal identification number allows data linkage across medical and administrative health care registries.

### Data Sources

The Danish Breast Cancer Group’s (DBCG) clinical database^32^ covers the entire Danish population with BC and includes clinical data on patients with early BC diagnosed in Denmark since 1977, with a completeness exceeding 95%.^33^ All hospital departments involved in BC diagnosis, treatment, and follow-up within the Danish health care system submit patient data to the DBCG on standardized forms.^34^ Detailed information on data sources is available in the eAppendix in Supplement 1.

### Study Population

**Figure 1.**
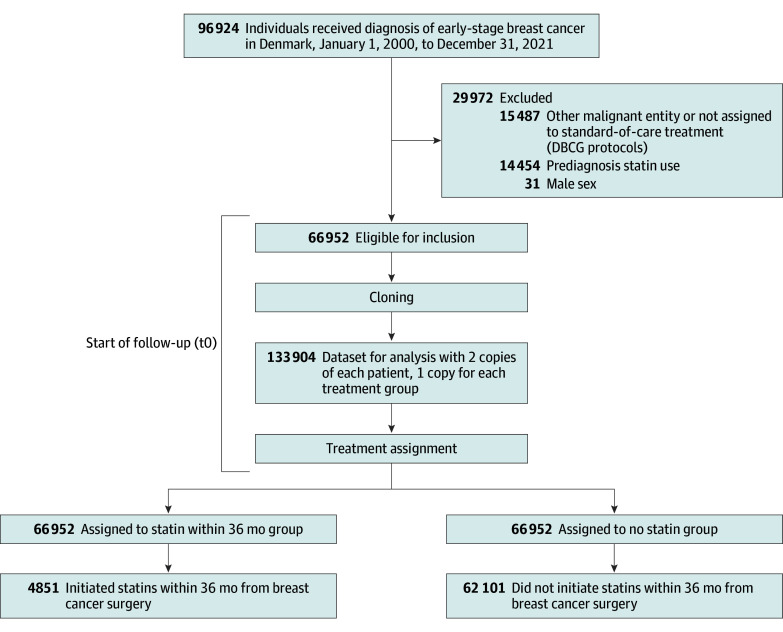
Flowchart of the Study Population DBCG indicates Danish Breast Cancer Group.

We identified 96 924 patients with early-stage BC diagnosed between 2000 and 2021 in Denmark and registered in the DBCG clinical database. We excluded 15 487 patients with other malignant neoplasms or who were not assigned to standard adjuvant therapy regimens, 31 male patients, and 14 454 patients with prediagnosis statin use. The final study population included 66 952 female patients with BC (Figure 1).

### Study Design

We aimed to emulate the ongoing MASTER trial^28^ by using prospectively collected observational data from the DBCG clinical database^34^ and health registry data. We aligned our emulated trial protocol with the design and specifications of the MASTER trial protocol (Table 1). In this emulation, we specified a protocol for statin initiation within 36 months after BC diagnosis and followed up patients until death or 10 years of follow-up. In the emulated trial, we included all Danish patients with BC who met the following eligibility criteria: (1) female sex; (2) aged 18 years or older; (3) diagnosis with stage I, II, or III invasive BC between the years 2000 and 2021; and (4) no prescription for any statin within 1 year before enrollment.

**Table 1.** Target Trial Protocol and Emulation

Study element	Trial	
	Target (ie, the MASTER trial)	Emulated
Eligibility criteria	(1) Female individual; (2) age ≥18 y; (3) diagnosed with stage I, II, or III breast cancer between the years 2000 and 2021; (4) no history of invasive breast carcinoma; (5) no prescription for any statin within 1 y before enrollment.	Same as for the target trial.
Treatment allocation	Patients were randomized to either (1) initiate statins within 36 mo following breast cancer diagnosis and stay on treatment for 2 y, or (2) not initiate statins.	Eligible patients who initiate statin treatment within 36 mo from diagnosis are included in the exposed group (1), and patients who do not initiate treatment within 36 mo from diagnosis are included in the unexposed group (2).
Follow-up period	Started at inclusion and ended at death, emigration, end of clinical follow-up, 10 y of follow-up, or October 5, 2022, whichever came first.	Same as for the target trial.
Outcomes	Breast cancer mortality and all-cause mortality.	Same as for the target trial.
Causal contrasts of interest	Per-protocol effect.	Per-protocol effect size adjusted for baseline covariates associated with the prognosis of breast cancer.
Analysis plan	Hazard ratio of breast cancer mortality and all-cause mortality. Five- and 10-y survival. In the analyses of breast cancer mortality, other causes of mortality were regarded as competing events.	Same as for the target trial.

### Treatment Strategies

We established 2 distinct exposure groups serving as treatment groups in the emulated trial. The following treatment strategy groups were compared: (1) initiation of statin therapy of any type and at any dosage within 36 months after BC diagnosis; and (2) no initiation of statin therapy within 36 months after BC diagnosis. Initiation of statin therapy was defined as dispensing at least 1 prescription after diagnosis within the 36-month grace period. Follow-up was restricted to periods of adherence to the assigned strategy during grace periods, as detailed in the Statistical Analysis section.

### Covariates

In the target trial emulation, the inverse probability of censoring weighting (IPCW) model included: age at diagnosis (continuous), year of diagnosis (continuous), tumor size (<10, 10–20, and >20 mm), nodal status (N0, N1–3, and ≥N4), Charlson Comorbidity Index score (0, 1–2, and ≥3), estrogen receptor status (positive or negative), type of primary surgery (mastectomy or breast-conserving), adjuvant radiotherapy (yes or no), adjuvant chemotherapy (yes or no), adjuvant endocrine therapy (yes or no), and cardiovascular medications (antiarrhythmics, antihypertensives, diuretics, vasodilators, β-blockers, renin–angiotensin–aldosterone system [RAAS] inhibitors, antiplatelets, anticoagulants, calcium channel blockers, and nitrates; all yes or no). The outcome model in the target trial emulation included no baseline covariates and was weighted by the IPCW. For the conventional time-varying Cox and landmark analyses, the outcome models were adjusted for the same set of variables.

### Follow-Up

The date of BC diagnosis served as time 0 of the follow-up period for each individual. This was the date when the eligibility criteria were met, and the individual was assigned to 1 of the treatment groups. Participants were then followed up from time 0 until death, emigration, end of clinical follow-up, 10 years of follow-up, or the end of follow-up on 5 October 2022, whichever occurred first.

### Outcomes

The primary end point was time to BC mortality, defined as the time from meeting the inclusion criteria until death from BC. The secondary end point was time to all-cause mortality, defined as the time from meeting the inclusion criteria until death from any cause.

### Statistical Analysis

To emulate a target trial of statin initiation after a BC diagnosis, we used a methodological framework previously described in detail by Maringe et al.^35^ First, we created a dataset with 2 copies of each eligible patient at baseline (BC diagnosis) and assigned each of the replicates to 1 of the 2 treatment strategies. Initiation of statin therapy was defined as dispensing at least 1 prescription after breast cancer diagnosis within the 36-month grace period. Replicates assigned to the statin strategy were artificially censored at 36 months if statin therapy had not been initiated by that time. Replicates assigned to the nonstatin strategy were censored if they initiated statin therapy at any time during the grace period. At baseline, we assumed that all patients were equally likely to be offered statins. Therefore, all patients entered both groups of the trial (both the statin group and nonstatin group) independently of their subsequent statin status (initiator or noninitiator); thus, 2 clones of each patient were created, and 1 clone entered each study group. This process enabled us to double the size of our dataset and make the study groups identical in terms of baseline demographics and biological and clinical characteristics.

To address informative censoring introduced by artificial censoring during follow-up, we applied IPCW. To compute the individual probability of remaining uncensored at each time point, we used a logistic regression model, with censoring treated as the event and covariates included to account for factors associated with censoring. The resulting probabilities from this model were then used to calculate IPCW weights. Adding the weights to the study population created a pseudo-population in which confounders were equally distributed between treatment groups.^36^

In the analysis model, we calculated hazard ratios (HRs) and 95% CIs by fitting IPCW Cox regression models with a robust variance estimator to compare the hazards of BC mortality and all-cause mortality in statin initiators vs noninitiators. We evaluated the proportional hazards assumption in the Cox regression models by visual inspection of log-log survival curves for each covariate. No systematic deviations from proportionality were observed. Only patients with complete data on all regressed variables were included in the analyses. We further computed 5-year and 10-year overall survival in percentages and 95% CIs, along with restricted mean survival and 95% CIs. In addition, we estimated 10-year breast cancer mortality and calculated the number needed to treat as the inverse of the absolute risk difference.

Secondary analyses included traditional multivariable Cox regression models with statin use modeled as a time-varying exposure and landmark analyses conducted at 36 months after diagnosis. For the landmark analyses, time 0 was reset at 36 months, and only patients alive and uncensored at that time were included. HRs with 95% CIs for BC mortality and all-cause mortality were then estimated. We further expanded our secondary analyses by applying the IPCW model to evaluate the timing of statin initiation. Specifically, we assessed the association between statin initiation and the end points using predefined grace periods of 12 and 24 months following breast cancer diagnosis.

To assess whether the outcomes of statin initiation were more pronounced in certain patient subgroups, we conducted a priori specified subgroup analyses according to menopausal status, tumor size, lymph node status, ER status, *ERBB2* status, surgery, and adjuvant treatment regimen.

To further explore the potential effect of statin initiation on cardiovascular disease, we conducted a post hoc analysis to evaluate cardiovascular mortality as an outcome, applying the same methodological framework as outlined previously. Analyses were completed in SAS software version 9.4 (SAS Institute).

## Results

We included 66 952 patients with early-stage BC in this study (17 152 [27.6%] were aged 50-59 years). Among the patients enrolled in our emulated trial, 4851 initiated statin use within 36 months after BC diagnosis. During 606 266 person-years of follow-up, 9130 patients died from BC, and 19 679 enrolled patients died from other causes; the remaining 47 183 patients were censored because of emigration, completion of 10 years of follow-up, or reaching the last day of follow-up on October 5, 2022. The mean (SD) follow-up period was 9.1 (5.5) years, and deviation from the emulated trial protocol was observed in 50 362 patients (75.2%) in the statin group and 4851 patients (7.3%) in the control group.

Patients with a statin prescription were generally older and tended to have smaller tumors with lower histological grades (Table 2). They were less likely to have BC that had spread to the lymph nodes, were more frequently diagnosed with ER-positive BC, and were more likely to have normal ERBB2 expression. As well, they were less likely to undergo mastectomy and chemotherapy, but were more often treated with radiotherapy and endocrine therapy.

**Table 2.** Baseline Patient and Treatment Characteristics According to Observed Statin Use Before Cloning

Characteristic	Patients, No. (%)		
	Statin prescription within 36 mo		Total
	No	Yes	
Total	62 101 (100.0)	4851 (100.0)	66 952 (100.0)
Age at diagnosis, y			
18-49	13 994 (22.5)	305 (6.3)	14 299 (21.4)
50-59	17 152 (27.6)	1206 (24.9)	18 358 (27.4)
60-69	16 761 (27.0)	1992 (41.1)	18 753 (28.0)
≥70	14 194 (22.9)	1348 (27.8)	15 542 (23.2)
Year of diagnosis			
2000-2004	15 323 (24.7)	809 (16.7)	16 132 (24.1)
2005-2009	13 158 (21.2)	1306 (26.9)	14 464 (21.6)
2010-2014	13 875 (22.3)	1017 (21.0)	14 892 (22.2)
2015-2018	10 937 (17.6)	928 (19.1)	11 865 (17.7)
2019-2021	8808 (14.2)	791 (16.3)	9599 (14.3)
Histological type			
No special type	49 131 (79.1)	3845 (79.3)	52 976 (79.1)
Lobular	6904 (11.1)	537 (11.1)	7441 (11.1)
Other	5477 (8.8)	450 (9.3)	5927 (8.9)
Missing	589 (0.9)	19 (0.4)	608 (0.9)
Histological grade			
Grade I	15 896 (25.6)	1459 (30.1)	17 355 (25.9)
Grade II	26 401 (42.5)	2086 (43.0)	28 487 (42.5)
Grade III	13 550 (21.8)	878 (18.1)	14 428 (21.5)
Missing/unclassified	6254 (10.1)	428 (8.8)	6682 (10.0)
Tumor size, mm			
<10	32 673 (52.6)	2828 (58.3)	35 501 (53.0)
10-20	21 544 (34.7)	1578 (32.5)	23 122 (34.5)
>20	1980 (3.2)	117 (2.4)	2097 (3.1)
Missing	5904 (9.5)	328 (6.8)	6232 (9.3)
Node status			
0	32 128 (51.7)	2829 (58.3)	34 957 (52.2)
1-3	16 428 (26.5)	1226 (25.3)	17 654 (26.4)
≥4	7310 (11.8)	466 (9.6)	7776 (11.6)
Missing	6235 (10.0)	330 (6.8)	6565 (9.8)
ER status			
ER-positive	52 641 (84.8)	4198 (86.5)	56 839 (84.9)
ER-negative	9460 (15.2)	653 (13.5)	10 113 (15.1)
ERBB2 status^a^			
Normal	35 839 (57.7)	3142 (64.8)	38 981 (58.2)
Overexpressed	6425 (10.3)	456 (9.4)	6881 (10.3)
Missing	19 837 (31.9)	1253 (25.8)	21 090 (31.5)
Breast surgical procedure			
Mastectomy	25 093 (40.4)	1706 (35.2)	26 799 (40.0)
Breast-conserving surgery	34 924 (56.2)	3070 (63.3)	37 994 (56.7)
Other	1054 (1.7)	26 (0.5)	1080 (1.6)
Missing	1030 (1.7)	49 (1.0)	1079 (1.6)
Chemotherapy			
No	37 442 (60.3)	3441 (70.9)	40 883 (61.1)
Yes	24 659 (39.7)	1410 (29.1)	26 069 (38.9)
Radiotherapy			
No	24 178 (38.9)	1635 (33.7)	25 813 (38.6)
Yes	37 923 (61.1)	3216 (66.3)	41 139 (61.4)
Endocrine therapy			
No	33 352 (53.7)	2364 (48.7)	35 716 (53.3)
Yes	28 749 (46.3)	2487 (51.3)	31 236 (46.7)

Abbreviations: ER, estrogen receptor; ERBB2, human epidermal growth factor receptor 2.

^a^ ERBB2 status was incompletely recorded during the early years of the study period, as routine testing was not clinically available until the revision of national guidelines in 2010.

**Figure 2.**
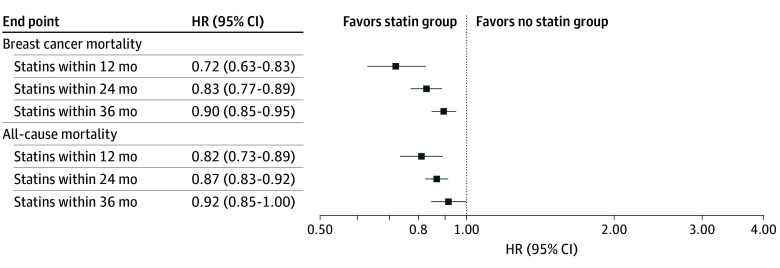
Hazard Ratios for Breast Cancer Mortality and All-Cause Mortality Among Statin Users Within 12-, 24-, and 36-Month Grace Periods From the Start of the Emulated Trial Results are presented with 95% CIs, demonstrating progressive attenuation of the protective association over longer inclusion periods. The figure displays an inverse probability of censoring weighting survival analyses of breast cancer mortality and all-cause mortality comparing the statin group with the no statin group in the cohort of patients diagnosed with early breast cancer between 2000 and 2021 who were registered in the Danish Breast Cancer Group registry using different grace periods (windows of inclusion). The displayed estimates include all patients with breast cancer in the study population irrespective of stage of disease and hormone receptor status.

The 10-year risk of BC mortality was 11.8% (95% CI, 10.8% to 12.7%) in the statin group and 13.5% (95% CI, 13.2% to 13.8%) in the control group, corresponding to a risk difference of 1.7% (95% CI, 0.5% to 3.0%) and a number needed to treat of 59 to prevent 1 additional breast cancer death; the HR was 0.90 (95% CI, 0.85 to 0.95) (Figure 2). A similar difference was observed for all-cause mortality (23.3% vs 24.5%; risk difference, 1.2%; 95% CI, –0.1% to 2.5%). The time-dependent Cox regression and the landmark analysis yielded greater effect estimates for statin initiators vs noninitiators (Cox HR, 0.79; 95% CI, 0.70 to 0.88; and landmark HR, 0.83; 95% CI, 0.73 to 0.95).

The 5-year risk difference was 2.0% (95% CI, 1.6%-2.4%) lower among statin initiators (89.8%) than among noninitiators (87.8%). The survival advantage persisted over a 10-year period, with survival rates of 76.7% for statin initiators and 75.5% for noninitiators. Additionally, the restricted mean survival time analysis found that statin initiators had slightly longer survival (8.9 years) than noninitiators (8.8 years) (Table 3).

**Table 3.** Overall Survival Outcomes in the Statin Group vs the Control Group

Outcome	Group, % (95% CI)		Difference, percentage points
	Statin	Control	
5-y Survival	89.8 (89.2 to 90.5)	87.8 (87.5 to 88.1)	2.0 (1.6 to 2.4)
10-y Survival	76.7 (75.5 to 78.0)	75.5 (75.1 to 75.9)	1.2 (−0.1 to 2.5)
Restricted mean survival time (95% CI), y	8.9 (8.7 to 9.1)	8.8 (8.7 to 8.9)	0.1 (−0.1 to 0.3)

We next conducted a secondary analysis using the same IPCW model to explore whether initiating statins closer to the BC diagnosis might have more beneficial effects (Figure 2). Patients who initiated statins within a 12-month window after BC diagnosis had a lower hazard of BC mortality (HR, 0.72; 95% CI, 0.63-0.83) and all-cause mortality (HR, 0.81; 95% CI, 0.73-0.89) than noninitiators. Similarly, BC mortality (HR, 0.83; 95% CI, 0.77-0.89) and all-cause mortality (HR, 0.87; 95% CI, 0.83-0.92) were lower for patients who initiated statins within 24 months after BC diagnosis than for noninitiators. IPCW analysis yielded an HR of 0.92 (95% CI, 0.85-1.00) for all-cause mortality among statin initiators vs noninitiators.

In the subgroup analyses of tumor and treatment characteristics conducted using IPCW models, we observed a reduction in BC mortality among statin initiators with node-positive BC (HR, 0.90; 95% CI, 0.85-0.96) (eTable 1 and eFigures 1 and 2 in Supplement 1). Furthermore, both patients with ER-positive and ER-negative BC appeared to benefit from statin use in terms of BC mortality, with HRs of 0.89 (95% CI, 0.83-0.96) and 0.92 (95% CI, 0.84-1.00), respectively. The association between statin initiation and BC mortality was also consistent for postmenopausal patients (HR, 0.90; 95% CI, 0.84-0.95) but not for premenopausal patients (HR, 0.90; 95% CI, 0.79-1.03).

Among patients who underwent mastectomy (HR, 0.91; 95% CI, 0.85-0.97) and patients who underwent breast-conserving surgery (HR, 0.90; 95% CI, 0.81-1.00), statin initiation was associated with lower BC mortality than that observed in noninitiators. The breast cancer mortality reduction associated with statin use was consistent regardless of whether patients were intended to receive radiation therapy (HR, 0.90; 95% CI, 0.84-0.97) or not (HR, 0.91; 95% CI, 0.84-0.99). Neither did the association differ by chemotherapy; an HR of 0.90 (95% CI, 0.82-0.98) was observed for statin initiators who were intended to receive chemotherapy, and a similar reduction was observed in patients who were not (HR, 0.90; 95% CI, 0.84-0.97).

Finally, among statin initiators who were not intended to receive endocrine therapy, a notable reduction in BC mortality was observed (HR, 0.90; 95% CI, 0.85-0.96). However, the precision of the effect size was low among those intended to receive endocrine therapy, and the 95% CI crossed the null (HR, 0.92; 95% CI, 0.81-1.04). The post hoc analysis produced a reduced hazard for cardiovascular mortality among statin initiators, but the 95% CI crossed the null (HR, 0.94; 95% CI, 0.84-1.05) (eTable 2 in Supplement 1).

## Discussion

Our cohort study showed that statin initiation within 36 months after diagnosis was associated with prolonged survival in patients with early-stage BC. Notably, the survival benefit was more pronounced among patients initiating statins closer to the time of diagnosis. These findings highlight the potential of statins as an adjunctive therapy in BC treatment, offering a promising avenue for enhancing clinical outcomes.

While earlier studies have indicated survival benefits of statins in patients with BC, concerns have been raised regarding the study designs used to address this question. Consequently, numerous calls^4,37,38^ have been made for randomized clinical trials evaluating the addition of statins to standard adjuvant therapy, thus prompting the initiation of the MASTER trial in 2020.^39^

In parallel with the trial’s launch, advanced methods for using observational data to emulate clinical trial conditions have become increasingly available. Because the primary results of the MASTER trial remain to be reported, our study was undertaken to simulate the trial and to evaluate the potential benefit of statins in patients with BC who are not currently eligible for inclusion, specifically those with ER-negative BC. In contrast to earlier studies^40,41,42^ reporting large decreases in BC mortality risk in patients using statins, our study suggested only a modest decrease in the risk of dying from BC among statin users.

### Limitations

This study has several limitations. First, when a patient was assigned to the statin group, we assumed that the patient continued to take statins throughout the follow-up period and did not account for statin discontinuation. Statins are prescribed for lifelong use. Discontinuation is often linked to mortality indicators (eg, metastatic disease, adverse effects, or contraindications).^24,43^ Yet, it is important to recognize that while statin adherence rates are high in the general population, they fall short of complete.^44^ Results should be interpreted considering the 36-month grace period. Associations appeared stronger with earlier statin initiation, as shown in secondary 12-month analyses. However, notably, this study aimed to emulate the MASTER trial, and hence a 36-month grace period was used. The study lacks data on lifestyle risk factors, such as body mass index, physical activity, and diet, potentially leading to residual and unmeasured confounding. If residual confounding were present, a risk of overestimating the effects of statins on all-cause mortality would exist (however, this does not appear to be the case, given the HR of 0.92), because statin users have a higher prevalence of all-cause mortality risk factors (eg, high cholesterol and heart disease) than nonusers. The effects of potential confounding on BC mortality are uncertain. In this study, competing events (ie, deaths from causes other than BC) were handled as censoring events. Additionally, this study is limited because the target trial emulation method applies the new-initiator design, which excludes all prevalent users of statins. This approach limits our understanding of how prediagnosis statin use may affect tumor phenotypes and other prognostic factors. Because statins are widely used, many breast cancer patients are not statin-naive at diagnosis.^45^

The main clinical message of our study is that statins may decrease the risk of dying from BC, but the true effect of statins on BC survival remains impossible to determine without a randomized clinical trial. The observed number needed to treat suggests a modest yet clinically meaningful benefit of statin therapy in this population. Despite the use of advanced design and analytic methods to eliminate common biases and confounding in pharmacoepidemiology, we cannot rule out the presence of residual bias in our study. The ongoing MASTER trial will provide a conclusive answer as to whether statins can be beneficially added to adjuvant treatment regimens for BC.

## Conclusions

Statins have considerable promise as adjuvant agents in BC therapy. Although further clinical trials will be crucial to validate these effects and to define optimal dosing and patient subgroups, a growing body of evidence highlights their potential as a valuable addition to the current arsenal of BC treatments.

## Notes

Supplement 1. eTable 1. Results of the IPCW, Cox Regression and Landmark Analysis Including the Prevalent Users of Statins eTable 2. Results of the Full Statistical Analysis Using a Tertiary Endpoint (Cardiovascular Mortality) eFigure 1. Inverse Probability-Weighted Kaplan-Meier Survival Curves for Breast Cancer–Specific Survival Among Statin Initiators and Noninitiators eFigure 2. Inverse Probability-Weighted Kaplan-Meier Survival Curves for Overall Survival Among Statin Initiators and Noninitiators eAppendix. Detailed Description of Data Sources eReferences

Supplement 2. Data Sharing Statement
