# Supplementary material for: Postdiagnosis Statin Use and Breast Cancer Mortality
Source: JAMA Netw Open. 2025 Oct 30;8(10):e2538737. doi: 10.1001/jamanetworkopen.2025.38737 (PMC12576488; doi:10.1001/jamanetworkopen.2025.38737)
Supplement: Supplement 1. — eTable 1. Results of the IPCW, Cox Regression and Landmark Analysis Including the Prevalent Users of Statins eTable 2. Results of the Full Statistical Analysis Using a Tertiary Endpoint (Cardiovascular Mortality) eFigure 1. Inverse Probability-Weighted Kaplan-Meier Survival Curves for Breast Cancer–Specific Survival Among Statin Initiators and Noninitiators eFigure 2. Inverse Probability-Weighted Kaplan-Meier Survival Curves for Overall Survival Among Statin Initiators and Noninitiators eAppendix. Detailed Description of Data Sources eReferences [file jamanetwopen-e2538737-s001.pdf]

Supplemental Online Content

Harborg S, Pedersen L, Sørensen HT, Ahern TP, Cronin-Fenton D, Borgquist S. Postdiagnosis statin use and breast cancer mortality. *JAMA Netw Open*. 2025;8(10):e2538737. doi:10.1001/jamanetworkopen.2025.38737

eTable 1. Results of the IPCW, Cox Regression and Landmark Analysis Including the Prevalent Users of Statins

eTable 2. Results of the Full Statistical Analysis Using a Tertiary Endpoint (Cardiovascular Mortality)

eFigure 1. Inverse Probability-Weighted Kaplan-Meier Survival Curves for Breast Cancer–Specific Survival Among Statin Initiators and Noninitiators

eFigure 2. Inverse Probability-Weighted Kaplan-Meier Survival Curves for Overall Survival Among Statin Initiators and Noninitiators

eAppendix. Detailed Description of Data Sources

eReferences

This supplemental material has been provided by the authors to give readers additional information about their work.

© 2025 Harborg S et al. *JAMA Network Open*.

eTable 1. Results of the IPCW, Cox Regression and Landmark Analysis Including the Prevalent Users of Statins (i.e. inclusion of the patients excluded during the wash-out period).

HZ_timedependent	HZ_landmark	HZ_cloning	outcome	exposure
0.84 (0.78-0.90)	0.84 (0.76-0.93)	1.06 (0.92-1.23)	Breast_cancer_death	Exposure_0_36_month
1.03 (0.92-1.15)	0.74 (0.62-0.88)	0.65 (0.44-0.97)	Cardiac_death	Exposure_0_36_month
0.86 (0.82-0.90)	0.84 (0.79-0.91)	0.68 (0.45-1.01)	Allcause_death	Exposure_0_36_month

eTable 2. Results of the Full Statistical Analysis Using a Tertiary Endpoint (Cardiovascular Mortality)

HZ_timedependent	HZ_landmark	HZ_cloning	outcome	exposure
1.44 (1.11-1.87)	1.14 (0.82-1.59)	0.85 (0.68-1.07)	Cardiac_death	Exposure_0_12_month
1.39 (1.14-1.69)	0.96 (0.74-1.25)	0.92 (0.80-1.05)	Cardiac_death	Exposure_0_24_month
1.32 (1.11-1.57)	0.95 (0.75-1.20)	0.94 (0.84-1.05)	Cardiac_death	Exposure_0_36_month

© 2025 Harborg S et al. *JAMA Network Open*.

eFigure 1. Inverse Probability-Weighted Kaplan-Meier Survival Curves for Breast Cancer–Specific Survival Among Statin Initiators and Noninitiators

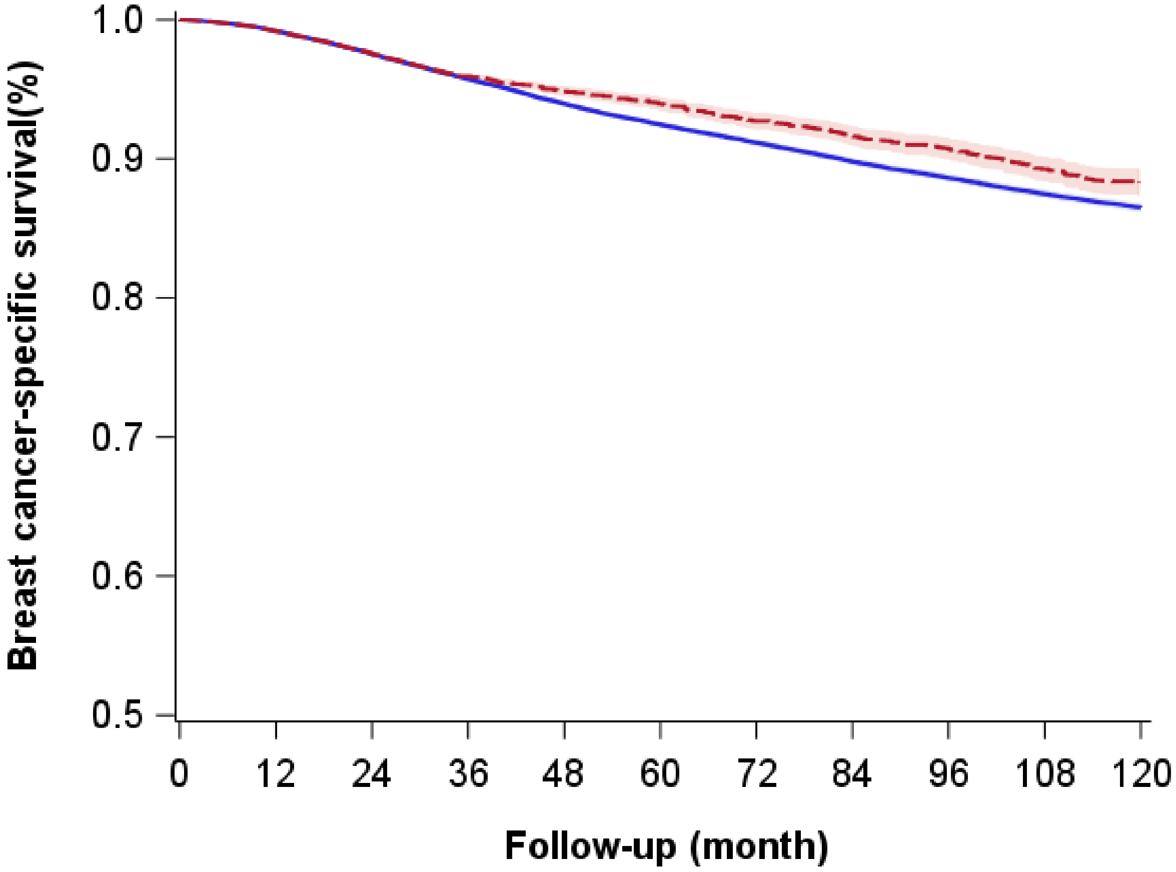

© 2025 Harborg S et al. *JAMA Network Open*.

eFigure 2. Inverse Probability-Weighted Kaplan-Meier Survival Curves for Overall Survival Among Statin Initiators and Noninitiators

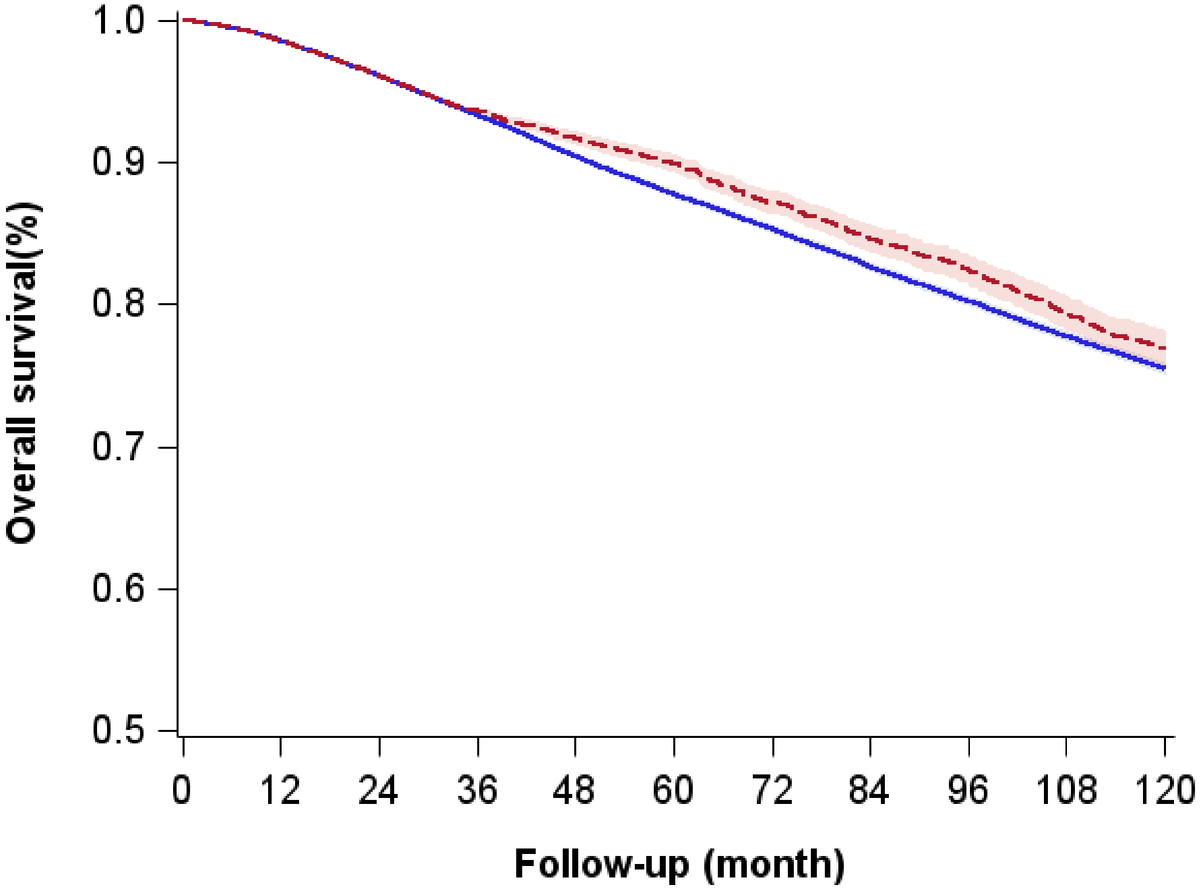

© 2025 Harborg S et al. *JAMA Network Open*.

eAppendix. Detailed Description of Data Sources

The Danish Breast Cancer Group's (DBCG) clinical database covers the entire Danish population with BC and includes clinical data on patients with early BC diagnosed in Denmark since 1977, with a completeness exceeding 95%.[1] The information retrieved from the DBCG registry for the current study included vital status, patient age at diagnosis, menopausal status, type of breast surgery, histologic tumor grade and type, nodal status, tumor size, estrogen receptor (ER) status, human epidermal growth factor receptor 2 (HER2) status, intention-to treat adjuvant radiotherapy, intention-to-treat adjuvant chemotherapy, and intention-to-treat adjuvant endocrine therapy.

The Danish Civil Registration System[2] has maintained data on the Danish population since 1968. This registry, updated daily, records each individual's civil registration number, date of birth, and information on vital and migration status.

The Danish National Patient Registry[3], established in 1977, contains comprehensive data on hospital admissions, discharges, emergency visits, and outpatient appointments. For each hospital visit, one primary diagnosis and up to 20 secondary diagnoses are recorded using the ICD-8 and 10 coding system.

To assess each patient's comorbid conditions, we used diagnoses recorded at the time of breast cancer diagnosis, applying the diseases in Charlson Comorbidity Index with exclusions for breast cancer and non-melanoma skin cancer. Additionally, the Danish National Prescription Registry[4], established in 1995, provided detailed records of filled prescriptions—including Anatomical Therapeutic Chemical (ATC) codes and dispensing dates from hospitals and pharmacies across Denmark. This data source allowed us to gather information on statin use to allocate treatment strategies, as well as data on the following medications: antiarrhythmics, antihypertensives, diuretics, vasodilators, beta-blockers, RAAS inhibitors, antithrombotics, anticoagulants, calcium channel inhibitors, and nitrates.

Since 1943, the Danish National Board of Health has maintained a comprehensive national registry of all deaths in Denmark, with records becoming individualized and digitized in 1970. In 1994, the Danish Registry of Causes of Death[5] adopted the ICD-10 coding system, adhering to the World Health Organization's guidelines for classifying causes of death. This registry records the date of death, along with the underlying and contributing causes as determined by the certifying physician. Leveraging this resource, we identified patients where breast cancer was recorded as the underlying cause of death, enabling reliable ascertainment of breast cancer-specific mortality within our study population.

All data sources described were linked at the individual level using the unique identifier assigned to all Danish residents at birth or immigration.

© 2025 Harborg S et al. *JAMA Network Open*.

eReferences

1. Cronin-Fenton DP, Kjærsgaard A, Ahern TP, et al. Validity of Danish Breast Cancer Group (DBCG) registry data used in the predictors of breast cancer recurrence (ProBeCaRe) premenopausal breast cancer cohort study. *Acta Oncol Stockh Swed*. 2017;56(9):1155-1160. doi:10.1080/0284186X.2017.1327720

2. Schmidt M, Pedersen L, Sorensen HT. The Danish Civil Registration System as a tool in epidemiology. *Eur J Epidemiol*. 2014;29(8):541-549. doi:10.1007/s10654-014-9930-3

3. The Danish National Patient Register - Elsebeth Lynge, Jakob Lynge Sandegaard, Matejka Rebolj, 2011. Accessed January 24, 2022. https://journals.sagepub.com/doi/10.1177/1403494811401482?url_ver=Z39.88-2003&rfr_id=ori:rid:crossref.org&rfr_dat=cr_pub%20%200pubmed

4. Pottegård A, Schmidt SAJ, Wallach-Kildemoes H, Sørensen HT, Hallas J, Schmidt M. Data Resource Profile: The Danish National Prescription Registry. *Int J Epidemiol*. 2017;46(3):798-798f. doi:10.1093/ije/dyw213

5. Helweg-Larsen K. The Danish Register of Causes of Death. *Scand J Public Health*. 2011;39(7_suppl):26-29. doi:10.1177/1403494811399958

© 2025 Harborg S et al. *JAMA Network Open*.
