# Supplementary material for: Postdiagnosis Statin Use and Breast Cancer Mortality
Source: JAMA Netw Open. 2025 Oct 30;8(10):e2538737. doi: 10.1001/jamanetworkopen.2025.38737 (PMC12576488; doi:10.1001/jamanetworkopen.2025.38737)
Supplement: Supplement 2. — Data Sharing Statement [file jamanetwopen-e2538737-s002.pdf]

Data Sharing Statement

Harborg. Postdiagnosis Statin Use and Breast Cancer Mortality. *JAMA Netw Open*. Published October 22, 2025. doi:10.1001/jamanetworkopen.2025.38737

Data
Data available: No

Additional Information
Explanation for why data not available: The data that support the findings of this study are available to academic researchers through project applications to the Danish Clinical Quality Program–National Clinical Registries (RKKP) at https://www.rkkp.dk/.
